# Variations in length of stay among survived very preterm infants admitted to Chinese neonatal intensive care units

**DOI:** 10.1007/s12519-021-00494-1

**Published:** 2022-01-05

**Authors:** Min Zhang, Yan-Chen Wang, Jin-Xing Feng, Ai-Zhen Yu, Jing-Wei Huang, Si-Yuan Jiang, Xin-Yue Gu, Jian-Hua Sun, Yun Cao, Wen-Hao Zhou, Shoo K. Lee, Li-Li Wang, Rong Yin, Chinese Neonatal Network

**Affiliations:** 1 Division of Neonatology, The First Affiliated Hospital of Anhui Medical University, No.218, Jixi Road, Shushan District, Hefei 230022, China; 2 NHC Key Laboratory of Neonatal Diseases, Fudan University, Children’s Hospital of Fudan University, Shanghai 201102, China; 3 Division of Neonatology, Shenzhen Children’s Hospital, Shenzhen 518038, China; 4 Division of Neonatology, Children’s Hospital of Fudan University, 399 Wanyuan Road, Minhang District, Shanghai 201102, China; 5 Division of Neonatology, Shanghai Children’s Medical Center, Shanghai Jiao Tong University School of Medicine, Shanghai 200127, China; 6 Maternal-Infants Care Research Centre and Department of Pediatrics, Mount Sinai Hospital, Toronto, ON M5G 1X5 Canada; 7 Professor Emeritus, University of Toronto, Toronto, ON M5T 3M7 Canada

**Keywords:** Length of stay to discharge home, Neonatal intensive care, Preterm, Risk factors

## Abstract

**Background:**

This study aimed to describe length of stay (LOS) to discharge and site variations among very preterm infants (VPIs) admitted to 57 Chinese neonatal intensive care units (NICUs) and to investigate factors associated with LOS for VPIs.

**Methods:**

This retrospective multicenter cohort study enrolled all infants < 32 weeks’ gestation and admitted to 57 NICUs which had participated in the Chinese Neonatal Network, within 7 days after birth in 2019. Exclusion criteria included major congenital anomalies, NICU deaths, discharge against medical advice, transfer to non-participating hospitals, and missing discharge date. Two multivariable linear models were used to estimate the association of infant characteristics and LOS.

**Results:**

A total of 6580 infants were included in our study. The overall median LOS was 46 days [interquartile range (IQR): 35–60], and the median corrected gestational age at discharge was 36 weeks (IQR: 35–38). LOS and corrected gestational age at discharge increased with decreasing gestational age. The median corrected gestational age at discharge for infants at 24 weeks, 25 weeks, 26 weeks, 27–28 weeks, and 29–31 weeks were 41 weeks, 39 weeks, 38 weeks, 37 weeks and 36 weeks, respectively. Significant site variation of LOS was identified with observed median LOS from 33 to 71 days in different hospitals.

**Conclusions:**

The study provided concurrent estimates of LOS for VPIs which survived in Chinese NICUs that could be used as references for medical staff and parents. Large variation of LOS independent of infant characteristics existed, indicating variation of care practices requiring further investigation and quality improvement.

**Supplementary Information:**

The online version contains supplementary material available at 10.1007/s12519-021-00494-1.

## Introduction

In the past two decades, the number of very preterm infants (VPIs, < 32 weeks’ gestation) who were cared for in Chinese neonatal intensive care units (NICU) has increased significantly with notably improved survival rates [1]. Therefore, quality of the comprehensive management of VPIs has been emphasized in recent years. Length of stay (LOS) is an important quality index that is associated with multiple factors, including infant characteristics and NICU management, as well as availability of post-discharge health care [2]. A LOS estimate is also an important piece of information for parents and medical staff of VPIs.

Prolonged LOS not only poses a heavy burden on perinatal healthcare, it also is related to increased exposure to adverse events, including infection, parent-infant separation, and sensory stimuli harmful to neurodevelopment [3–5]. On the other hand, if discharge occurs before the establishment of physiologic stability, increased risk of life-threatening events and re-hospitalization have been observed [6, 7]. Currently, there are no national or international guidelines on optimal LOS for VPIs. Several studies have reported hospital-level, regional or international variations of LOS among this high-risk population, indicating varying care practice, organizational structures and policies [8–11].

To our knowledge, there is no multicenter study in China for evaluating the current LOS in Chinese NICUs. National-level references for Chinese medical staff and parents are lacking to estimate and to benchmark LOS among survived VPIs who are discharged home. Our study used 2019 data from the Chinese Neonatal Network (CHNN). We aimed to describe LOS of surviving VPIs cared for in 57 tertiary NICUs across China, and to investigate factors associated with LOS to discharge home.

## Methods

### Chinese Neonatal Network and participating hospitals

This cohort study was based on the data collected from the CHNN, a national network of Chinese tertiary NICUs. Participating NICUs of CHNN have recognized expertise in caring for high-risk neonates in China. CHNN has created and organized a standardized clinical database of VPI to benchmark outcomes and care practices since January, 2019. In 2019, 57 hospitals collected whole-year data in CHNN database [12]. These hospitals included 4 national children’s medical centers, 4 regional children’s medical centers, 30 provincial perinatal or children’s medical centers, and 19 hospitals as major referral centers in large cities. 42 hospitals were perinatal centers with birthing facilities, and 15 hospitals were free-standing children’s hospitals that only admitted outborn infants. All the hospitals had ability to provide complicate care for infants < 32 weeks’ gestation.

### Study population

All infants born less than 32 weeks of gestational age and admitted to CHNN NICUs within 7 days after birth from January 2019 to December 2019 were included. Exclusion criteria included major congenital anomalies, NICU deaths, infants who were discharged against medical advice, infants transferred to non-participating hospitals within 24 hours after birth, and infants with missing date of discharge. Transfers between participating hospitals were tracked as data from the same infants until NICU discharge.

### Data collection

Detailed clinical data were collected prospectively by trained data abstractors using a standardized database in each hospital. Data were entered directly into a customized database with built-in error checking and a standard manual of operations and definitions. Data were transmitted electronically to the CHNN coordinating center in Children’s Hospital of Fudan University. Checks for data quality were then conducted quarterly, and wrong data were sent back and corrected at each site. A data audit was conducted periodically to ensure consistency of data abstraction [13].

### Definitions

Gestational age was determined using the hierarchy of best estimate based on prenatal ultrasound, menstrual history, obstetric examination, or postnatal estimate of gestation using the Ballard score [14]. Small for gestational age (SGA) was defined as birth weight < 10th percentile for the gestational age according to the Chinese neonatal birth weight values [15]. Usage of antenatal corticosteroids was defined as maternal receipt of at least one dose of dexamethasone or betamethasone before delivery, including complete and incomplete administration. Intraventricular hemorrhage (IVH) was defined using Papile’s criteria [16]. Cystic periventricular leukomalacia (PVL) was defined as the presence of periventricular cysts on cranial ultrasound or magnetic resonance imaging. Necrotizing enterocolitis (NEC) was defined as stage II two or above according to Bell’s criteria [17, 18]. Bronchopulmonary dysplasia (BPD) was defined as ventilation or oxygen dependency at 36 weeks’ postmenstrual age [19]. Severe retinopathy of prematurity (ROP) was diagnosed according to the international classification of retinopathy of prematurity [20]. Sepsis was defined as positive blood or cerebrospinal fluid culture and antibiotic therapy or intent of antibiotics therapy ≥ 5 days [21]. Transport risk index of physiologic stability (TRIPS) score was used to indicate illness severity on admission [22, 23].

### Statistical analysis

Baseline characteristics and LOS were summarized descriptively. The categorical characteristics were summarized as absolute numbers with percentages and compared using *χ*^2^ test. For quantitative baseline characteristics, normally distributed variables were expressed as means with standard deviation, and highly skewed variables were expressed as median with interquartile range. Student’s *t* test for normally distributed variables or Wilcoxon test for highly skewed variables were applied when comparing characteristics between infants admitted to different types of hospitals.

To determine the factors associated with length of stay, a univariate linear model was applied. Model parameters were estimated using a generalized estimating equation (GEE) to account for cluster effects of infants within CHNN sites. Since LOS was not normally distributed, LOS was log-transformed before using regression analyses. Two multivariable linear regression models with a GEE approach were applied to estimate the adjusted association between LOS and each factor. In model 1, perinatal factors were adjusted, including maternal age, primigravida, maternal hypertension, maternal diabetes, antenatal corticosteroids, C-section, gestational age, SGA, infant sex, multiple birth, inborn status, Apgar score ≤ 7 at 5 minutes and TRIPS score. To assess the effect of neonatal morbidities on LOS, severe IVH, NEC, BPD, severe ROP and sepsis were included in model 2 in addition to the factors included in model 1. Geometric mean ratios of LOS were calculated from the above-mentioned models to express the association of each factor with LOS. Geometric mean ratios of LOS were calculated by dividing the mean of LOS in the exposure group by the mean of LOS in the un-exposure group. Sensitivity analyses were performed separately for perinatal centers and free-standing children’s hospitals. ﻿The median LOS in different hospitals was compared. To account for different characteristics of infants admitted to different hospitals, adjusted LOS in each hospital was estimated by the multivariable models 1 and 2 mentioned earlier.

SAS Version 9.4 (SAS Institute, Cary, North Carolina) was used for data management and data analysis. A two-tailed test with significance level (*P* < 0.05) was applied in our study.

### Research ethical approval

Ethical approval was obtained from the Ethics Review Board of Children’s Hospital of Fudan University (2018-296), which was recognized by all participating hospitals. Waiver of consent was granted at all sites owing to our use of de-identified data.

## Results

### Study population

**Fig. 1:**
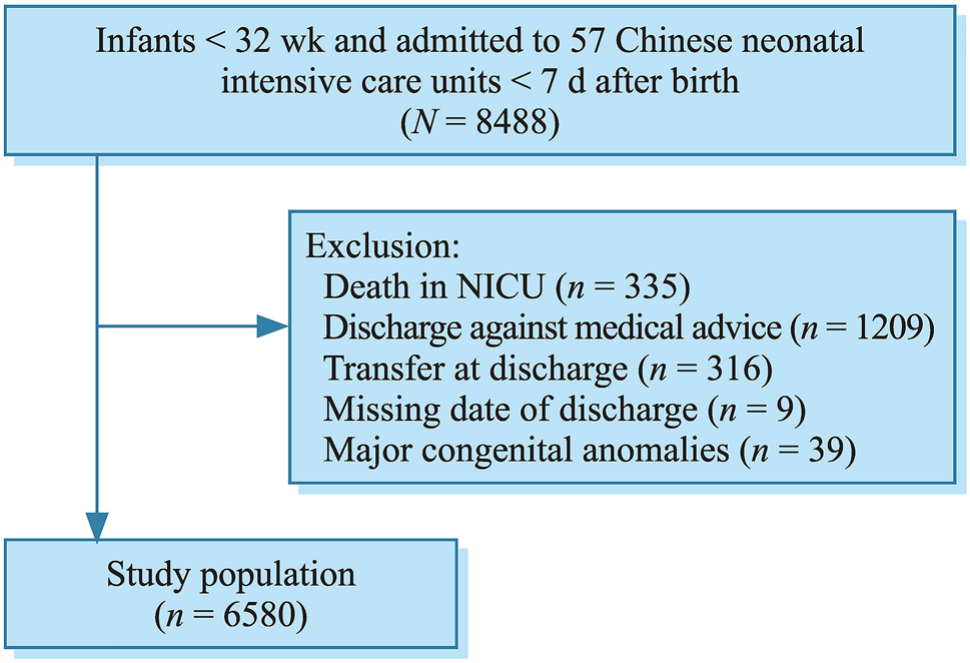
Study population. *NICU* neonatal intensive care unit

In the 57 participating hospitals, 8488 VPIs were admitted to NICUs within 7 days after birth in 2019. Of these infants, there were 1908 infants excluded in this study because of deaths, discharge against medical advice, transfers, major congenital anomalies, and missing date of discharge. The remaining 6580 VPIs that survived to discharge were included in the final analysis (Fig. 1).

The baseline characteristics of VPIs enrolled in our study are provided in Table 1. The median of gestational age of enrolled infants was 30.0 weeks (28.9–31.0), and birth weight was 1366.6 ± 303.0 g. A total of 791 (12.0%) infants were < 28 weeks, and 5789 (88.0%) infants were of gestational age 28–31 weeks. Overall, 2292 (34.8%) surviving infants had any major morbidities during hospitalization.

**Table 1:** Baseline characteristic of very preterm infants survived to discharge in Chinese neonatal intensive care units

Characteristics	< 28 wk	28–31 wk	Total
Number of infants	791	5789	6580
Maternal characteristics			
Maternal age (y), mean ± SD	31.89 ± 4.67	31.11 ± 4.88	31.20 ± 4.87
Primigravida, *n*/*N* (%)	428/784 (54.6)	2929/5757 (50.9)	3357/6541 (51.3)
Maternal hypertension, *n*/*N* (%)	80/782 (10.2)	1174/5727 (20.5)	1254/6509 (19.3)
Maternal diabetes, *n*/*N* (%)	138/780 (17.7)	1059/5723 (18.5)	1197/6503 (18.4)
Antenatal corticosteroids, *n*/*N* (%)	588/733 (80.2)	4412/5439 (81.1)	5000/6172 (81.0)
C-section, *n*/*N* (%)	245/788 (31.1)	3570/5768 (61.9)	3815/6556 (58.2)
Infant characteristics			
Gestational age (wk), median (IQR)	27.1 (26.3–27.6)	30.29 (29.3–31.1)	30.00 (28.9–31.0)
≤ 25, *n*/*N* (%)	108/791 (13.7)	–	108/6580 (1.6)
26–27, *n*/*N* (%)	683/791 (86.3)	–	683/6580 (10.4)
28–29, *n*/*N* (%)	–	2194/5789 (37.9)	2194/6580 (33.3)
30–31, *n*/*N* (%)	–	3595/5789 (62.1)	3595/6580 (54.6)
Birth weight (kg), mean ± SD	994.5 ± 169.6	1417.4 ± 280.9	1366.6 ± 303.0
Small for gestational age, *n*/*N* (%)	10/791 (1.3)	398/5784 (6.9)	408/6575 (6.2)
Male, *n*/*N* (%)	468/791 (59.2)	3236/5784 (55.9)	3704/6575 (56.3)
Multiple birth, *n*/*N* (%)	252/791 (31.9)	1702/5789 (29.4)	1954/6580 (29.7)
Inborn, *n*/*N* (%)	558/791 (70.5)	4135/5789 (71.4)	4693/6580 (71.3)
Apgar score ≤ 7 at 5 min, *n*/*N* (%)	83/749 (11.1)	228/5538 (4.1)	311/6287 (4.9)
TRIPS score, median (IQR)	19 (12–22)	12 (6–19)	12 (6–19)
Major infant morbidities			
Any morbidities, *n*/*N* (%)	473/791 (59.8)	1819/5789 (31.4)	2292/6580 (34.8)
IVH grade III and above or cPVL, *n*/*N* (%)^a^	116/738 (15.7)	422/5254 (8.0)	538/5992 (9.0)
NEC ≥ stage II, *n*/*N* (%)	45/791 (5.7)	205/5789 (3.5)	250/6580 (3.8)
BPD, *n*/*N* (%)	349/791 (44.1)	1057/5789 (18.3)	1406/6580 (21.4)
Severe ROP, *n*/*N* (%)^b^	74/772 (9.6)	104/5019 (2.1)	178/5791 (3.1)
Sepsis, *n*/*N* (%)	111/791 (14.0)	464/5789 (8.0)	575/6580 (8.7)

*IQR* interquartile range, *SD* standard deviation, *TRIPS* transport risk index of physiologic stability, *IVH* intraventricular hemorrhage, *cPVL* cystic periventricular leukomalacia, *NEC* necrotizing enterocolitis, *BPD* bronchopulmonary dysplasia, *ROP* retinopathy of prematurity. ^a^Incidence of IVH grade III and above or cPVL was calculated within infants who had neuroimaging results; ^b^incidence of ROP was calculated within infants who finished the ROP screening. “–” no data

### Length of stay across gestational age

The LOS for neonates and the corrected gestational age at discharge are shown by each week of gestational age in Table 2. The median LOS for all VPIs was 46 (35–60) days, and the corrected gestational age at discharge was 36 (35–38) weeks. LOS decreased with increasing gestational age. The corrected gestational age also decreased with increasing gestational age. The median corrected gestational age at discharge for infants at 24 weeks, 25 weeks, 26 weeks, 27 weeks and 28 weeks were 41 (39–42) weeks, 39 (37–42) weeks, 38 (37–40) weeks, 37 (36–39) weeks and 37 (36–38) weeks, respectively. Infants born at 29–31 weeks were discharged at a median corrected gestational age of 36 weeks.

**Table 2:** Length of stay and corrected gestational age at discharge for very preterm infants survived to discharge in Chinese neonatal intensive care units

GA (wk)	Number of infants	LOS (d), median (IQR)	cGA at discharge (wk), median (IQR)
< 24	2	121 (107–135)	41 (39–43)
24	18	112 (104–125)	41 (39–42)
25	88	95 (82–116)	39 (37–42)
26	211	82 (72–93)	38 (37–40)
27	472	69 (60–82)	37 (36–39)
28	910	60 (50–70)	37 (36–38)
29	1284	50 (42–59)	36 (35–38)
30	1625	41 (33–51)	36 (35–38)
31	1970	34 (27–43)	36 (35–37)
Total	6580	46 (35–60)	36 (35–38)

*LOS* length of stay, *IQR* interquartile range, *GA* gestational age, *cGA* corrected gestational age

### Factors associated with length of stay

Factors associated with LOS are shown in Table 3. In univariable analyses, maternal age, primigravida, maternal hypertension, decreasing gestational age, SGA, Apgar score ≤ 7 at 5 minutes, higher TRIPS score, and all types of major morbidities were associated with increasing LOS.

**Table 3:** Factors associated with length of stay among very preterm infants survived to discharge in Chinese neonatal intensive care units

Factors	Unadjusted mean ratio^a^	Adjusted mean ratio 1^a,b^	Adjusted mean ratio 2^a,c^
Maternal characteristics			
Maternal age	1.01 (1.00, 1.03)	1.00 (0.99, 1.01)	1.00 (0.99, 1.01)
Primigravida	1.03 (1.01, 1.06)	1.02 (1.00, 1.03)	1.02 (1.00, 1.03)
Maternal hypertension	1.07 (1.04, 1.10)	1.07 (1.05, 1.09)	1.06 (1.04, 1.08)
Maternal diabetes	1.01 (0.98, 1.05)	0.99 (0.97, 1.02)	0.99 (0.97, 1.01)
Antenatal corticosteroids	0.98 (0.95, 1.01)	0.99 (0.98, 1.01)	0.99 (0.98, 1.01)
C-section	0.94 (0.91, 0.97)	1.03 (1.01, 1.05)	1.02 (1.01, 1.04)
Infant characteristics			
Gestational age (wk)			
< 24	2.65 (2.58, 2.72)	2.63 (2.53, 2.73)	2.41 (2.34, 2.48)
24	3.09 (2.91, 3.27)	3.15 (2.97, 3.33)	2.53 (2.33, 2.76)
25	2.69 (2.50, 2.89)	2.81 (2.63, 2.99)	2.36 (2.26, 2.47)
26	2.17 (2.08, 2.26)	2.23 (2.16, 2.31)	2.02 (1.97, 2.07)
27	1.91 (1.84, 1.99)	1.97 (1.91, 2.04)	1.86 (1.81, 1.91)
28	1.64 (1.59, 1.70)	1.70 (1.65, 1.75)	1.62 (1.58, 1.66)
29	1.37 (1.33, 1.42)	1.42 (1.38, 1.46)	1.38 (1.35, 1.41)
30	1.17 (1.14, 1.20)	1.18 (1.16, 1.21)	1.17 (1.15, 1.19)
31	Reference	Reference	Reference
Small for gestational age	1.25 (1.21, 1.30)	1.40 (1.36, 1.44)	1.33 (1.29, 1.37)
Male	1.00 (0.98, 1.02)	0.98 (0.97, 1.00)	0.97 (0.96, 0.99)
Multiple birth	0.99 (0.97, 1.02)	1.01 (0.99, 1.03)	1.00 (0.99, 1.02)
Inborn	0.96 (0.93, 1.00)	0.96 (0.93, 1.00)	0.98 (0.95, 1.02)
Apgar score ≤ 7 at 5 min	1.21 (1.16, 1.27)	1.02 (0.99, 1.05)	1.00 (0.98, 1.02)
TRIPS score	1.07 (1.06, 1.08)	1.02 (1.02, 1.03)	1.02 (1.01, 1.02)
Infant morbidities			
IVH grade III and above or cPVL	1.19 (1.14, 1.24)		1.05 (1.03, 1.07)
NEC ≥ stage II	1.29 (1.22, 1.36)		1.19 (1.15, 1.24)
BPD	1.52 (1.46, 1.58)		1.25 (1.23, 1.28)
Severe ROP	1.46 (1.33, 1.60)		1.09 (1.05, 1.14)
Sepsis	1.26 (1.21, 1.31)		1.11 (1.08, 1.15)

Generalized linear models with generalized estimating equation were applied to account for the cluster effects in Chinese Neonatal Network. *TRIPS* transport risk index of physiologic stability, *IVH* intraventricular hemorrhage, *cPVL* cystic periventricular leukomalacia, *NEC* necrotizing enterocolitis, *BPD* bronchopulmonary dysplasia, *ROP* retinopathy of prematurity. ^a^Geometric mean ratio were reported; ^b^adjustment for model 1 including: maternal age, primigravida, maternal hypertension, maternal diabetes, antenatal corticosteroids, C-section, gestational age, small for gestational age, infant sex, multiple birth, inborn status, Apgar score ≤ 7 at 5 min and TRIPS score; ^c^adjustment for model 2 including: factors adjusted in model 1 plus severe IVH or cPVL, NEC, BPD, severe ROP and sepsis

In multivariable model 1, which included only perinatal factors, after adjustment, primigravida, maternal hypertension, cesarean section, decreasing gestational age, SGA and higher TRIPS scores remained significantly related to increasing LOS, with the highest adjusted mean ratios for gestational age and SGA.

Multivariable model 2 also included neonatal morbidities. The relationship between perinatal factors and LOS remained similar with model 1. Each type of major morbidities was positively associated with increasing LOS, showing BPD with adjusted mean ratio 1.25 (1.23–1.28), NEC > stage II with adjusted mean ratio 1.19 (1.15–1.24), sepsis with adjusted mean ratio 1.11 (1.08–1.15), severe ROP with adjusted mean ratio 1.09 (1.05–1.14) and IVH grade III and above or cystic PVL with adjusted mean ratio 1.05 (1.03–1.07).

Sensitivity analysis in the subgroup of perinatal centers and free-standing children’s hospitals showed consistent associations between LOS and maternal hypertension, gestational age, SGA, TRIPS score and neonatal morbidities (Supplementary Tables 1, 2).

### Variations of length of stay among different neonatal intensive care units

**Fig. 2:**
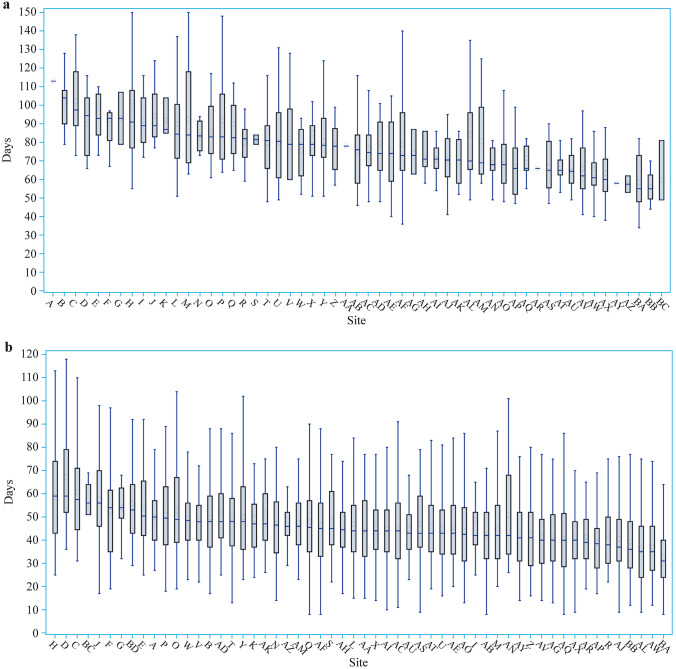
Distribution of LOS for very preterm infants across Chinese Neonatal Network sites. **a** Distribution of LOS for infants less than 28 weeks’ gestation; **b** distribution of LOS for infants at 28–31 weeks’ gestation. *LOS* length of stay

**Fig. 3:**
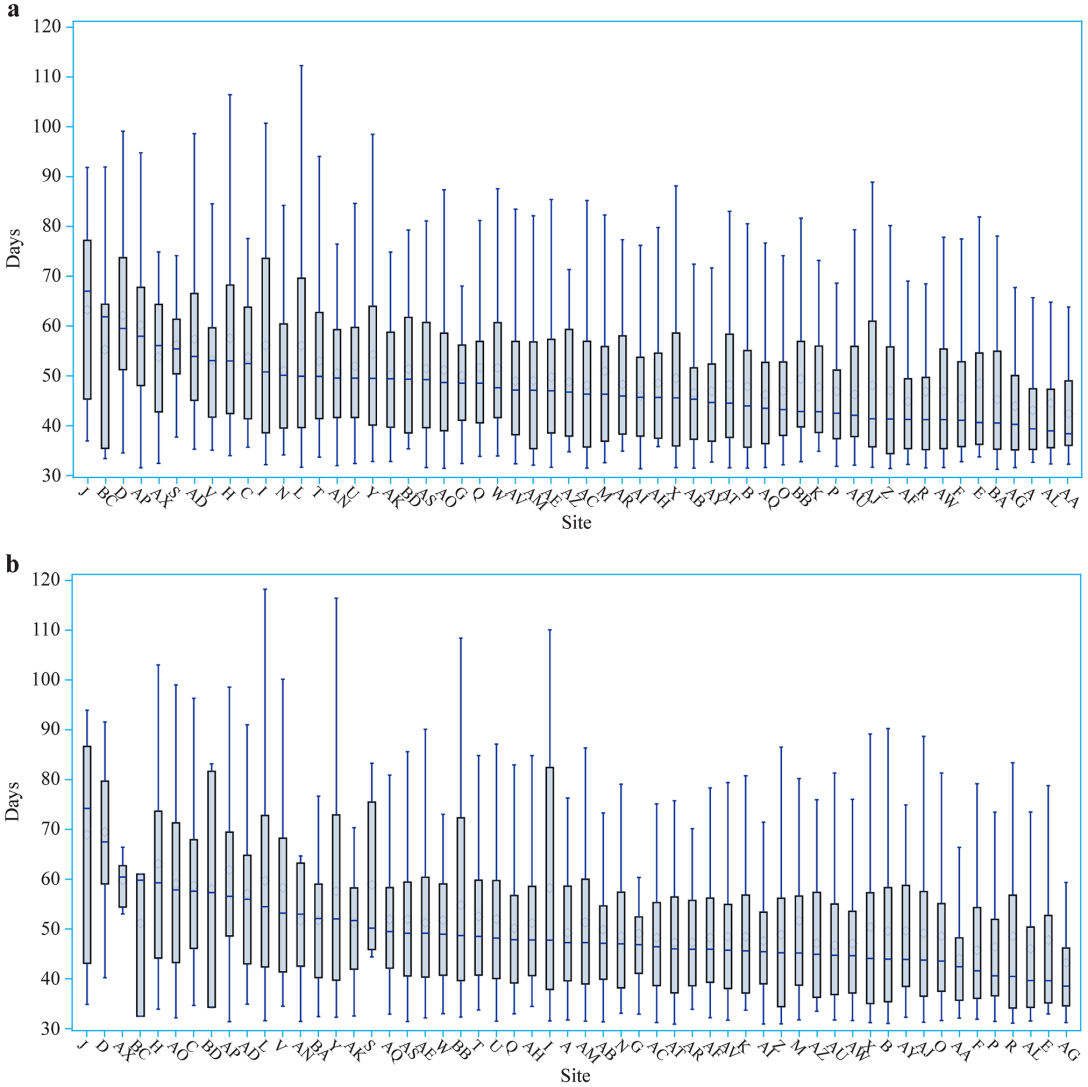
Adjusted LOS for very preterm infants across Chinese Neonatal Network sites. **a** Site variation of adjusted LOS adjusting for prenatal factors included in model 1; **b** site variation of adjusted LOS adjusting for factors included in model 2. *LOS* length of stay

The median LOS (*P* < 0.01) was significantly longer at children’s hospitals than at prenatal centers (Supplementary Table 1). The observed median LOS for surviving VPIs in 57 NICUs is presented in Fig. 2. For infants < 28 weeks, the median LOS varied from 49 (49–81) to 113 (113–113) in different NICUs, and there was no significant difference (*P* = 0.21) of LOS among NICUs with different numbers of annual admissions of infants < 28 weeks (Supplementary Table 3). For infants born at 28–31 weeks, the median LOS varied from 31 (24–40) to 59 (43–74). There was a trend of increasing LOS as numbers annual admissions decreased (*P* < 0.01, Supplementary Table 3). After adjustment of perinatal factors, the variation of LOS remained significant (Fig. 3a). Even after adjustment of neonatal morbidities, the median adjusted LOS varied by 1.94 times among different NICUs (Fig. 3b).

## Discussion

To our knowledge, our study is the first national-level investigation focusing on LOS among surviving VPIs who were discharged home from Chinese NICUs. Our results will provide essential data for parents to estimate LOS of their VPIs and for medical staff to benchmark. Significant variations on LOS are identified among different Chinese NICUs, which could not be explained by heterogeneity of the admission population.

The median LOS for VPIs in our study was 46 days. When gestational age increased by 1 week, the median LOS decreased by about 10 days. For infants at 27–28 weeks and 29–31 weeks, the median corrected gestational age at discharge were 37 weeks and 36 weeks, respectively. These data may be valuable in allowing medical staff and parents to predict approximately when their VPIs will be discharged while ensuring adequate and timely preparation for discharge. Historically, medical staff would use the estimated date of delivery as the likely discharge data to inform parents of VPIs [24]. Our data showed that most VPIs would go home 3–4 weeks earlier than the estimated date of delivery, consistent with the international trend of shorter LOS for VPIs [24, 25]. Information on possible discharge dates would improve parental understanding the course of NICU stay for their babies and preparation for discharge [26]. Therefore, more accurate data of LOS may benefit medical staff to estimate and prepare for discharge. What is more, the development of a simple centile chart for LOS and dates of discharge at each gestation based on our national-level data may promote greater evidence-based communication with parents [24].

Comparing to other countries, LOSs among infants at 29–31 weeks from our study were similar to those reported from national data in England [25]. While for infants ≤ 28 weeks, our LOS was approximately 10 days shorter compared with those reported by England and other 11 developed countries [9, 25]. One possible explanation for shorter LOS in China may be relatively lower survival rates of infants with the smallest gestational age [27]. Greater survival in developed countries may result in more infants with morbidities associated with prolonged LOS. Certainly, there might be a different discharge threshold between China and other countries, and more investigation is needed to identify the potential for better practices. Another possible explanation might be efforts to shorten LOS in Chinese NICUs. Benefits of shorter LOS, such as reduced healthcare cost, may need to be carefully balanced with safe care management at home or in the community [28].

Significant variation existed in LOS among participating NICUs. The variation persisted after adjustment for important neonatal characteristics affecting LOS, in keeping with previous studies from other countries [9, 10]. Longer LOS was observed in Children’s hospitals compared with prenatal centers, possibly due to more complicated condition of VPIs admitted to children’s hospitals. This may reflect the differences in clinical care practices, healthcare policies and availability of proper post-discharge healthcare service. Such variation urges national guidelines suggesting current potential best criteria and discharge preparation for VPIs in China, and indicating opportunities for improving and standardizing care.

Our study revealed several perinatal factors that were associated with LOS. Besides gestational age, infants’ characteristics including small for gestational and increased disease severity on admission were significantly related with increased LOS. This finding is similar with previously studies and is consistent with clinical experience [9, 10]. Perinatal factors, such as primigravida, maternal hypertension, and C-section also were found to be related with LOS; however, their impacts were minimal.

More importantly, our study showed that each neonatal morbidity increased LOS among VPIs, with the largest effect coming from NEC and BPD. In our cohort, more than 1/3 of survived VPIs experienced at least one major morbidity. Therefore, when more VPIs survived, focus should be shifted to morbidity-free survival, which will result in shorter NICU stay and better long-term outcomes.

Our study has several strengths. This study is the first to report LOS of VPIs in China, which fills a data gap. Large sample size enabled us to present LOS at each gestation. Detailed data collection and strict data quality control make the examination of various related factors of LOS possible. Fifty-seven hospitals were from all major regions of China, representing a concurrent national-level situation. There are also several limitations in our study. First, all hospitals in our study had large tertiary neonatal service; therefore, our data could not represent the situation in lower level units. Second, care practices related with discharge planning in different hospitals were not collected, so further investigation on the reasons for site variation could not be performed. Third, information on socioeconomic forces was not collected.

In conclusion, our study provided current estimates of LOS for VPIs that survived in Chinese NICUs, which could be used as references for medical staff and parents. Infants at 27–31 weeks were likely to be discharged at a median of 36–37 weeks’ corrected gestational age. Increasing maternal age, primigravida, maternal hypertension, decreasing gestational age, SGA, Apgar score ≤ 7 at 5 minutes, higher TRIPS score, and all types of major morbidities were significantly associated with longer LOS. A large variation of LOS to discharge home of infant characteristics existed, indicating variation of care practices requiring further investigation and quality improvement.

## Supplementary Information

Below is the link to the electronic supplementary material.

Supplementary file 1 (DOCX 26 KB)

## Data Availability

The original contributions presented in the study were included in the article and supplementary materials. Further inquiries can be directed to the corresponding author.
