# Supplementary material for: Variations in length of stay among survived very preterm infants admitted to Chinese neonatal intensive care units
Source: World J Pediatr. 2022 Jan 5;18(2):126–34. doi: 10.1007/s12519-021-00494-1 (PMC8844160; doi:10.1007/s12519-021-00494-1)
Supplement: Supplementary file 1 — (DOCX 26 KB) [file 12519_2021_494_MOESM1_ESM.docx]

**Supplemental Table 1.** Baseline characteristic of very preterm infants survived to discharge admitted to different types of hospitals in China

| **Characteristics** | **Children's hospital (15 hospitals)** | **Prenatal center (42 hospitals)** |
| --- | --- | --- |
| **Number of infants** | 1222 | 5358 |
| **Length of stay, median (IQR)** | 51 (40-67) | 45 (34-59) |
| **Maternal characteristics** |  |  |
| Maternal age (y), mean ± SD | 31.12 ± 4.89 | 31.22 ± 4.86 |
| Primigravida, *n/N* (%) | 607/1218 (49.8) | 2750/5323 (51.7) |
| Maternal hypertension, *n/N* (%) | 183/1193 (15.3) | 1071/5316 (20.1) |
| Maternal diabetes, *n/N* (%) | 193/1188 (16.2) | 1004/5315 (18.9) |
| Antenatal corticosteroids, *n/N* (%) | 795/1036 (76.7) | 4205/5136 (81.9) |
| C-section, *n/N* (%) | 703/1210 (58.1) | 3112/5346 (58.2) |
| **Infant characteristics** |  |  |
| Gestational age (wk), median (IQR) | 30.0 (28.7-31.0) | 30.1 (29.0-31.0) |
| ≤ 25, *n/N* (%) | 15/1222 (1.2) | 93/5358 (1.7) |
| 26-27, *n/N* (%) | 140/1222 (11.5) | 543/5358 (10.1) |
| 28-29, *n/N* (%) | 434/1222 (35.5) | 1760/5358 (32.8) |
| 30-31, *n/N* (%) | 633/1222 (51.8) | 2962/5358 (55.3) |
| Birth weight (kg), mean ± SD | 1359.4 ± 298.7 | 1368.2 ± 303.9 |
| Small for gestational age, *n/N* (%) | 66/1222 (5.4) | 342/5353 (6.4) |
| Male, *n/N* (%) | 691/1222 (56.5) | 3013/5353 (56.3) |
| Multiple birth, *n/N* (%) | 365/1222 (29.9) | 1589/5358 (29.7) |
| Inborn, *n/N* (%) | 0/1222 (0.0) | 4693/5358 (87.6) |
| Apgar score ≤ 7 at 5 min, *n/N* (%) | 103/1140 (9.0) | 208/5147 (4.0) |
| TRIPS score, median (IQR) | 14 (8-21) | 12 (6-19) |
| **Major infant morbidities** |  |  |
| Any morbidities, *n/N* (%) | 501/1222 (41.0) | 1791/5358 (33.4) |
| IVH grade III and above or cPVL, *n/N* (%)^a^ | 117/1144 (10.2) | 421/4848 (8.7) |
| NEC ≥ stage II, *n/N* (%) | 66/1222 (5.4) | 184/5358 (3.4) |
| BPD, *n/N* (%) | 334/1222 (27.3) | 1072/5358 (20.0) |
| Severe ROP, *n/N* (%)^b^ | 56/1170 (4.8) | 122/4621 (2.6) |
| Sepsis, *n/N* (%) | 113/1222 (9.2) | 462/5358 (8.6) |

*IQR* interquartile range, *SD* standard deviation, *TRIPS* transport risk index of physiologic stability, *IVH* intraventricular hemorrhage, *cPVL* cystic periventricular leukomalacia, *NEC* necrotizing [enterocolitis](https://cn.bing.com/search?q=Enterocolitis&filters=sid%3a564dc4a8-4e51-3d9c-87f6-2c7f41da41e3&form=ENTLNK), *BPD* bronchopulmonary dysplasia, *ROP* [retinopathy](https://cn.bing.com/search?q=Diabetic+Retinopathy&filters=sid%3a502dad87-3760-4f25-997c-e2d1e94dac75&form=ENTLNK) of prematurity. ^a^Incidence of IVH grade III and above or cPVL was calculated within infants who had neuroimaging results; ^b^incidence of ROP was calculated within Infants who finished the ROP screening

**Supplemental Table 2.** Factors associated with length of stay among very preterm infants survived to discharge in different types of hospitals in China

| **Factors** | **Children's hospital** | | |  | **Prenatal center** | | |
| --- | --- | --- | --- | --- | --- | --- | --- |
|  | **Unadjusted mean ratio^a^** | **Adjusted mean ratio 1^a,b^** | **Adjusted mean ratio 2^a,c^** |  | **Unadjusted mean ratio^a^** | **Adjusted mean ratio 1^a,b^** | **Adjusted mean ratio 2^a,c^** |
| **Maternal characteristics** |  |  |  |  |  |  |  |
| Maternal age | 1.02 (0.99, 1.05) | 1.01 (0.99, 1.02) | 1.00 (0.99, 1.02) |  | 1.01 (1.00, 1.03) | 1.00 (0.99, 1.01) | 1.00 (0.99, 1.01) |
| Primigravida | 1.03 (0.99, 1.09) | 1.03 (0.99, 1.07) | 1.03 (1.00, 1.07) |  | 1.03 (1.01, 1.06) | 1.01 (1.00, 1.03) | 1.01 (0.99, 1.03) |
| Maternal hypertension | 1.05 (0.99, 1.11) | 1.05 (1.01, 1.10) | 1.05 (1.00, 1.11) |  | 1.07 (1.04, 1.10) | 1.07 (1.05, 1.10) | 1.06 (1.04, 1.09) |
| Maternal diabetes | 1.02 (0.96, 1.08) | 1.00 (0.96, 1.05) | 1.00 (0.96, 1.03) |  | 1.01 (0.97, 1.05) | 0.99 (0.97, 1.02) | 0.99 (0.97, 1.02) |
| Antenatal corticosteroids | 1.02 (0.94, 1.10) | 1.01 (0.96, 1.06) | 1.01 (0.96, 1.06) |  | 0.97 (0.94, 1.00) | 0.99 (0.97, 1.00) | 0.99 (0.97, 1.00) |
| C-section | 0.93 (0.90, 0.97) | 1.03 (0.99, 1.08) | 1.02 (0.99, 1.06) |  | 0.94 (0.91, 0.98) | 1.03 (1.00, 1.05) | 1.02 (1.00, 1.04) |
| **Infant characteristics** |  |  |  |  |  |  |  |
| Gestational age (wk) |  |  |  |  |  |  |  |
| < 24^d^ | N/A | N/A | N/A |  | 2.68 (2.60, 2.77) | 2.62 (2.51, 2.73) | 2.42 (2.33, 2.51) |
| 24 | 2.82 (2.76, 2.89) | 2.79 (2.58, 3.01) | 2.31 (2.19, 2.44) |  | 3.11 (2.92, 3.32) | 3.19 (3.01, 3.38) | 2.55 (2.33, 2.79) |
| 25 | 3.11 (2.65, 3.66) | 3.10 (2.54, 3.79) | 2.46 (2.18, 2.78) |  | 2.66 (2.46, 2.88) | 2.79 (2.61, 2.99) | 2.36 (2.25, 2.48) |
| 26 | 2.15 (2.00, 2.31) | 2.15 (1.99, 2.34) | 1.96 (1.82, 2.10) |  | 2.17 (2.07, 2.28) | 2.25 (2.17, 2.33) | 2.03 (1.97, 2.09) |
| 27 | 1.90 (1.79, 2.02) | 1.92 (1.82, 2.04) | 1.81 (1.72, 1.91) |  | 1.91 (1.83, 2.00) | 1.98 (1.91, 2.06) | 1.87 (1.81, 1.93) |
| 28 | 1.61 (1.55, 1.67) | 1.63 (1.57, 1.71) | 1.58 (1.52, 1.64) |  | 1.65 (1.58, 1.72) | 1.72 (1.66, 1.78) | 1.63 (1.58, 1.67) |
| 29 | 1.38 (1.30, 1.45) | 1.39 (1.33, 1.45) | 1.36 (1.31, 1.41) |  | 1.37 (1.32, 1.43) | 1.42 (1.38, 1.47) | 1.38 (1.34, 1.42) |
| 30 | 1.16 (1.11, 1.20) | 1.16 (1.12, 1.21) | 1.15 (1.12, 1.20) |  | 1.17 (1.13, 1.21) | 1.18 (1.16, 1.21) | 1.17 (1.15, 1.20) |
| 31 | Reference | Reference | Reference |  | Reference | Reference | Reference |
| Small for gestational age | 1.16 (1.09, 1.23) | 1.30 (1.22, 1.38) | 1.27 (1.19, 1.35) |  | 1.27 (1.22, 1.32) | 1.42 (1.37, 1.46) | 1.34 (1.30, 1.38) |
| Male | 1.04 (1.00, 1.09) | 1.01 (0.97, 1.05) | 1.00 (0.96, 1.03) |  | 0.99 (0.97, 1.01) | 0.98 (0.96, 0.99) | 0.97 (0.96, 0.98) |
| Multiple birth | 0.99 (0.93, 1.05) | 1.01 (0.98, 1.04) | 1.00 (0.98, 1.02) |  | 1.00 (0.97, 1.02) | 1.01 (0.99, 1.03) | 1.00 (0.98, 1.02) |
| Inborn^d^ | N/A | N/A | N/A |  | 1.00 (0.96, 1.04) | 0.99 (0.95, 1.03) | 1.00 (0.97, 1.04) |
| Apgar score ≤ 7 at 5 min | 1.17 (1.06, 1.29) | 1.02 (0.97, 1.06) | 1.00 (0.97, 1.02) |  | 1.23 (1.17, 1.29) | 1.03 (0.99, 1.06) | 1.00 (0.97, 1.03) |
| TRIPS score | 1.06 (1.05, 1.08) | 1.02 (1.01, 1.03) | 1.02 (1.01, 1.03) |  | 1.07 (1.06, 1.08) | 1.02 (1.02, 1.03) | 1.01 (1.01, 1.02) |
| **Infant morbidities** |  |  |  |  |  |  |  |
| IVH grade III and above or cPVL | 1.16 (1.06, 1.28) |  | 1.04 (1.01, 1.08) |  | 1.19 (1.14, 1.25) |  | 1.05 (1.02, 1.07) |
| NEC ≥ stage II | 1.35 (1.21, 1.50) |  | 1.16 (1.07, 1.25) |  | 1.27 (1.19, 1.35) |  | 1.21 (1.16, 1.26) |
| BPD | 1.42 (1.32, 1.52) |  | 1.22 (1.17, 1.27) |  | 1.54 (1.47, 1.61) |  | 1.26 (1.23, 1.29) |
| Severe ROP | 1.48 (1.29, 1.70) |  | 1.08 (1.03, 1.12) |  | 1.44 (1.29, 1.62) |  | 1.10 (1.05, 1.16) |
| Sepsis | 1.25 (1.16, 1.33) |  | 1.13 (1.07, 1.20) |  | 1.27 (1.21, 1.33) |  | 1.11 (1.07, 1.15) |

Generalized linear models with generalized estimating equation were applied to account for the cluster effects in Chinese Neonatal Network. *TRIPS* transport risk index of physiologic stability, *IVH* intraventricular hemorrhage, *cPVL* cystic periventricular leukomalacia, *NEC* necrotizing [enterocolitis](https://cn.bing.com/search?q=Enterocolitis&filters=sid%3a564dc4a8-4e51-3d9c-87f6-2c7f41da41e3&form=ENTLNK), *BPD* bronchopulmonary dysplasia, *ROP* [retinopathy](https://cn.bing.com/search?q=Diabetic+Retinopathy&filters=sid%3a502dad87-3760-4f25-997c-e2d1e94dac75&form=ENTLNK) of prematurity. ^a^Geometric mean ratio were reported; ^b^adjustment for model 1 including: maternal age, primigravida, maternal hypertension, maternal diabetes, antenatal corticosteroids, C-section, gestational age, small for gestational age, infant sex, multiple birth, inborn status, Apgar score ≤ 7 at 5 minutes and TRIPS score; ^c^adjustment for model 2 including: factors adjusted in model 1 plus severe IVH or cPVL, NEC, BPD, Severe ROP and sepsis; ^d^N/A means no infants in this categories

**Supplemental Table 3.** Length of stay and corrected gestational age at discharge for very preterm infants admitted to neonatal intensive care units with different number of annual admissions

| **Number of admission per year** | **Number of infants** | **LOS (d),** | **cGA at discharge (wk),** |
| --- | --- | --- | --- |
|  |  | **median (IQR)** | **median (IQR)** |
| **GA < 28 wk** |  |  |  |
| 0-10 | 111 | 81 (67-93) | 39 (37-40) |
| 11-20 | 244 | 75 (65-90.5) | 37 (36-40) |
| > 20 | 436 | 76 (65-90) | 38 (36-39) |
| **GA (28-31 wk)** |  |  |  |
| 0-50 | 424 | 47 (36-61) | 37 (36-38) |
| 51-100 | 1476 | 46 (36-57) | 37 (36-38) |
| 101-150 | 861 | 43 (33-54) | 36 (35-38) |
| 151-200 | 1130 | 42 (32-54) | 36 (35-38) |
| > 200 | 1898 | 43 (33-53) | 36 (35-37) |

*LOS* length of stay, *IQR* interquartile range, *GA* gestational age, *cGA* corrected gestational age
